# How Biologics Are Changing the Global Burden of Inflammatory Bowel Disease: A Review

**DOI:** 10.7759/cureus.112332

**Published:** 2026-07-09

**Authors:** Yousef Abu Osba, Sanad Elshebli, Abdullah Abureesh, Araek Al-Shraideh, Yousef Yousef, Mohammad Abu-Shaban, Omar Abureesh

**Affiliations:** 1 General Surgery, University Hospitals of Leicester NHS Trust, Leicester, GBR; 2 Trauma and Orthopedics, Buckinghamshire Healthcare NHS Trust, Buckinghamshire, GBR; 3 Medicine, University of Jordan, Amman, JOR; 4 Pediatrics, Staten Island University Hospital, Staten Island, USA; 5 Internal Medicine, Staten Island University Hospital, Staten Island, USA; 6 Internal Medicine, Southeast Georgia Health System, Brunswick, USA

**Keywords:** biologic therapy, biosimilars, crohn's disease, global health burden, inflammatory bowel disease, interleukin-23, tumor necrosis factor inhibitors, ulcerative colitis, ustekinumab, vedolizumab

## Abstract

Inflammatory bowel disease (IBD), comprising Crohn’s disease and ulcerative colitis, has evolved from a condition concentrated in high-income Western nations into a global disease. The therapeutic landscape has been transformed by biologic agents, including anti-tumor necrosis factor (anti-TNF) antibodies, anti-integrin therapy (vedolizumab), interleukin (IL)-12/23 blockade (ustekinumab), and selective IL-23 (p19) inhibitors (risankizumab and mirikizumab); more recently, oral small-molecule advanced therapies (Janus kinase (JAK) inhibitors and sphingosine-1-phosphate (S1P) receptor modulators) have broadened the treatment armamentarium further. This narrative review synthesizes contemporary epidemiological data such as incidence, prevalence, mortality, disability, healthcare utilization, and economic burden, and landmark randomized controlled trials (RCTs) to examine how biologic therapy is reshaping the global burden of IBD. We summarize the mechanisms and pivotal efficacy evidence for each major class, and we appraise their measurable impact on hard outcomes, mucosal healing, surgery, hospitalization, disability, and quality of life, alongside the comparative-effectiveness data emerging from head-to-head trials. We then consider an apparent paradox: although biologics reduce complications in trial settings, population-level reductions in surgery and hospitalization have been inconsistent, and the cost of IBD care has shifted toward pharmaceuticals and risen overall. Finally, we address the role of biosimilars in improving affordability and the persistent access divide between high-income and resource-limited settings. Because much of the population-level evidence is ecological or observational, associations between biologic use and improvements in mortality and disability should be interpreted with caution and cannot be regarded as proof of direct causation. Biologics have unquestionably improved the lives of individual patients; whether they reduce the global burden of IBD will depend on equitable access, optimal positioning, and integration with treat-to-target strategies.

## Introduction and background

Inflammatory bowel disease (IBD) encompasses two principal chronic, immune-mediated disorders of the gastrointestinal tract, Crohn’s disease and ulcerative colitis, characterized by relapsing and remitting inflammation, progressive bowel damage, and a substantial impact on quality of life. Once regarded as a disease of affluent, industrialized Western societies, IBD has become a global health concern. In a systematic review of population-based studies, incidence has stabilized in many Western countries while prevalence has surpassed 0.3% of the population, and incidence is accelerating in newly industrialized nations whose populations have adopted increasingly westernized lifestyles [1]. The Global Burden of Disease (GBD) studies have quantified this trajectory: the GBD 2017 analysis estimated that 6.8 million people were living with IBD worldwide, an increase of more than 80% since 1990, with years lived with disability roughly doubling over the same interval [2]. The GBD 2021 update reported a global age-standardized incidence rate that rose modestly from 4.22 to 4.45 per 100,000 person-years between 1990 and 2021, while age-standardized mortality declined from 0.60 to 0.52 per 100,000 [3].

Kaplan and Windsor have framed this global evolution as a sequence of epidemiological stages: emergence in regions of low incidence, acceleration in incidence, compounding prevalence in established high-prevalence regions, and a hypothesized future stage of prevalence equilibrium [4]. The practical consequence is that health systems on every continent must now plan for the delivery of complex, long-term IBD care; the analysis by Ananthakrishnan and colleagues of sustaining healthcare delivery for a growing, prevalent population explicitly makes this case [5]. It is against this backdrop of an expanding and aging prevalent population that the therapeutic revolution of the past quarter-century must be understood.

The introduction of monoclonal antibodies targeting specific effectors of mucosal inflammation has fundamentally altered the management of moderate-to-severe IBD. Where therapy was once limited to aminosalicylates, corticosteroids, and conventional immunomodulators, clinicians now select among several biologic classes with distinct mechanisms of action and safety profiles [6,7]. Two concepts recur throughout this review and merit brief definition for non-specialist readers. Mucosal healing (also termed endoscopic healing) refers to the resolution of visible inflammation and ulceration of the intestinal lining at endoscopy; it is a more stringent target than symptom control and correlates with reduced relapse, hospitalization, and surgery. Treat-to-target strategies use objective markers of inflammation, rather than symptoms alone, as explicit therapeutic goals, escalating therapy until targets such as clinical remission and mucosal healing are achieved.

Previous reviews have described the efficacy of individual biologic classes in IBD. The specific contribution of the present review is to connect that clinical-trial evidence explicitly to the population-level burden of disease incidence, prevalence, mortality, disability, healthcare utilization, and cost, and to examine the gap between individual efficacy and population impact, including the roles of biosimilars and global access. This review therefore addresses a deceptively simple question: are biologics changing the global burden of IBD? To answer it, we proceed as follows: we first outline the epidemiological context and the shifting global geography of IBD; we then summarize the mechanistic rationale and pivotal efficacy evidence for each biologic class and for emerging oral advanced therapies; we appraise the measurable effect of these agents on patient-important and health-system outcomes; and we finally consider the economic, biosimilar, and access factors that determine whether individual efficacy translates into a population-level reduction in burden.

## Review

Methods of literature retrieval

This is a narrative review, and the literature selection is therefore interpretive rather than governed by a predefined systematic protocol. 

Databases and dates

We searched Medical Literature Analysis and Retrieval System Online (MEDLINE)/PubMed, supplemented by the reference lists of major society guidelines and GBD publications, for English-language articles addressing the epidemiology of IBD and the efficacy, comparative effectiveness, safety, and health-economic impact of biologic therapies. Searches were performed from database inception and were last updated in May 2026; no lower date limit was applied to landmark trials.

Search terms

The core search combined disease terms (“inflammatory bowel disease” OR “Crohn disease” OR “ulcerative colitis”) with therapy and outcome terms (biologic, “tumor necrosis factor”, infliximab, adalimumab, golimumab, certolizumab, vedolizumab, ustekinumab, risankizumab, mirikizumab, “interleukin-23”, integrin, biosimilar) and epidemiological/health-system terms (epidemiology, “global burden”, incidence, prevalence, surgery, hospitalization, “quality of life”, cost, remission, “randomized controlled trial”). The complete database-specific strings are reproduced in Appendix A.

Study selection and prioritization

Priority was given, in a pre-specified hierarchy, to (i) phase 2/3 landmark randomized controlled trials (RCTs) underpinning regulatory approval, (ii) population-based epidemiological analyses and GBD studies, (iii) head-to-head comparative trials, and (iv) authoritative systematic reviews and consensus statements. Records were screened by two authors independently; candidate studies were compared against this hierarchy, and disagreements about inclusion or emphasis were resolved by discussion and, where necessary, adjudication by a third senior author. Because this is a narrative synthesis rather than a systematic review, we did not maintain a formal PRISMA flow diagram, and we did not record the exact number of records retrieved and screened; formal risk-of-bias scoring and quantitative meta-analysis were likewise not undertaken. We minimized selection bias by pre-specifying the prioritization hierarchy, screening in duplicate, deliberately seeking evidence that complicates the efficacy narrative (for example, cost-of-illness data and analyses showing no population-level fall in surgery), and cross-checking each cited study against its primary publication. These methodological constraints are acknowledged explicitly in the Limitations section.

The shifting global epidemiology of IBD

**Figure 1:**
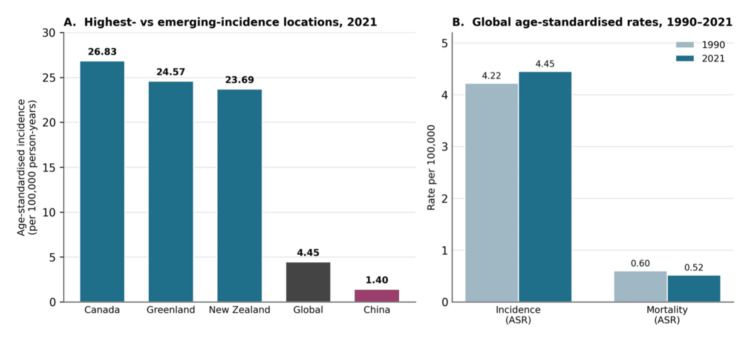
Global epidemiology of inflammatory bowel disease. (A) Age-standardized incidence rates per 100,000 person-years in 2021 for selected high-incidence locations compared with the global average and an emerging-incidence country (China). (B) Global age-standardized incidence and mortality rates in 1990 versus 2021, illustrating modestly rising incidence alongside falling mortality. ASR: age-standardized rate Data derived from Global Burden of Disease 2021 analyses [3]. This figure was created by the authors using Python version 3.10 with the Matplotlib library (Python Software Foundation, Wilmington, DE, USA).

The geography of IBD is in flux. The highest age-standardized incidence rates in 2021 were recorded in high-income regions-for example, Canada, Greenland, and New Zealand each reported incidence above 23 per 100,000-while many regions historically considered low-risk are experiencing rapid increases [3]. China exemplifies this transition, with incidence rising from well below one to approximately 1.4 per 100,000 and an estimated annual percentage change far exceeding the global average [3]. This pattern reflects the epidemiological-stage model: established high-prevalence regions face the compounding burden of a large, aging prevalent population, whereas emerging regions confront a steep rise in newly diagnosed cases (Figure 1) [1,4].

Two implications follow. First, the absolute number of people requiring sophisticated, often biologic-based, care is increasing worldwide. Second, the burden is shifting toward health systems-particularly in low- and middle-income countries-that are least prepared to finance high-cost therapies [5]. Any assessment of how biologics affect the global burden of IBD must therefore weigh demonstrable clinical efficacy against the realities of access and affordability.

Pathophysiological rationale for biologic therapy

**Figure 2:**
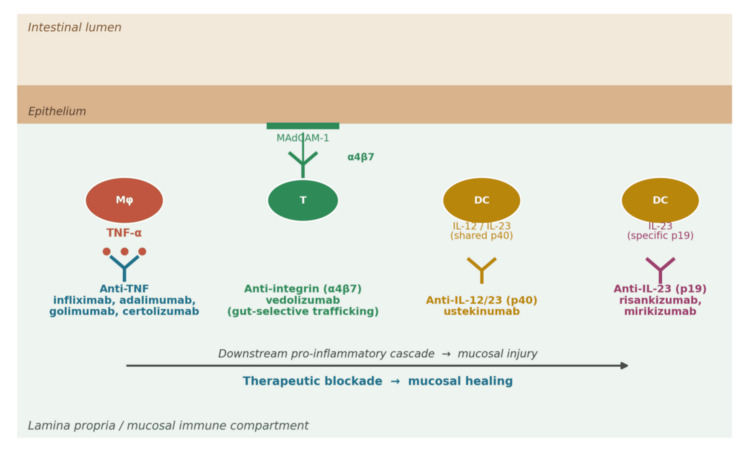
Principal molecular targets of biologic therapies in inflammatory bowel disease (IBD) Anti-TNF agents neutralize tumor necrosis factor-alpha; vedolizumab blocks the gut-selective α4β7 integrin–MAdCAM-1 interaction that governs lymphocyte homing; ustekinumab targets the shared p40 subunit of IL-12 and IL-23; and risankizumab and mirikizumab selectively inhibit the p19 subunit of IL-23. Mφ: macrophage; DC: dendritic cell; T: T lymphocyte; Anti-TNF: anti-tumor necrosis factor; Anti-IL: anti-interleukin This figure was created by the authors using Python version 3.10 with the Matplotlib library (Python Software Foundation, Wilmington, DE, USA).

IBD arises from a dysregulated mucosal immune response to luminal and microbial antigens in genetically susceptible hosts, with a breakdown of intestinal barrier integrity and amplification of pro-inflammatory cytokine networks [8]. Tumor necrosis factor-alpha (TNF-α), produced by activated macrophages and T cells, is a central effector cytokine driving epithelial injury, leukocyte recruitment, and granuloma formation. The interleukin (IL)-23/Th17 axis sustains chronic mucosal inflammation, and the trafficking of gut-homing lymphocytes to the intestine is mediated by the α4β7 integrin-MAdCAM-1 interaction [8]. These nodes define the principal druggable targets exploited by current biologics (Figure 2): neutralization of TNF-α, selective blockade of α4β7-mediated trafficking, and inhibition of the IL-12/IL-23 pathway, either at the shared p40 subunit or at the IL-23-specific p19 subunit. Intracellular signaling pathways downstream of several of these cytokines, in particular the Janus kinase (JAK)-STAT pathway and the sphingosine-1-phosphate (S1P) axis that regulates lymphocyte egress from lymph nodes, constitute additional druggable targets that are now addressed by oral small molecules.

Classes of biologic agents and landmark efficacy evidence

**Figure 3:**
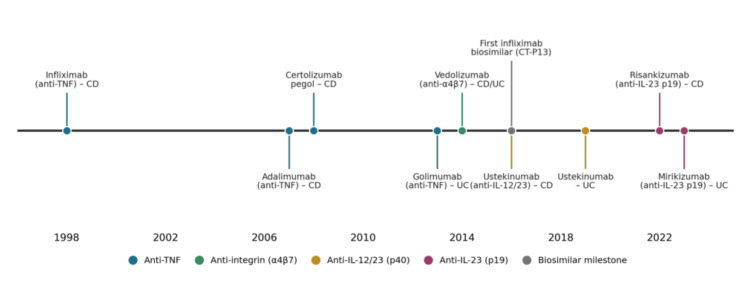
Approximate chronology of the introduction of biologic agents for inflammatory bowel disease, color-coded by mechanistic class, together with the emergence of the first infliximab biosimilar. Approval years are indicative of the broad sequence of availability and may vary by regulatory jurisdiction. CD: Crohn's disease; UC: ulcerative colitis; Anti-TNF-α: anti-tumor necrosis factor-alpha; Anti-IL: anti-interleukin This figure was created by the authors using Python version 3.10 with the Matplotlib library (Python Software Foundation, Wilmington, DE, USA).

The sequence of regulatory approvals over the past 25 years reflects the progressive translation of this mechanistic understanding into therapy (Figure 3). Table 1 summarizes the principal classes, their molecular targets, and their approved indications.

**Table 1:** Principal classes of biologic therapy used in inflammatory bowel disease (IBD). Approved indications are indicative and vary by regulatory jurisdiction and over time. Anti-TNF-α: anti-tumor necrosis factor-alpha; CD: Crohn’s disease; UC: ulcerative colitis; IV: intravenous; SC: subcutaneous; Anti-IL: anti-interleukin

Class/target	Representative agents	Route	Typical IBD indication	Mechanistic note
Anti-TNF-α	Infliximab, adalimumab, golimumab, certolizumab pegol	IV / SC	CD and UC (golimumab UC only; certolizumab CD)	Neutralize soluble and membrane-bound TNF-α
Anti-integrin (α4β7)	Vedolizumab	IV / SC	CD and UC	Gut-selective blockade of lymphocyte trafficking
Anti-IL-12/23 (p40)	Ustekinumab	IV then SC	CD and UC	Blocks shared p40 subunit of IL-12 and IL-23
Anti-IL-23 (p19)	Risankizumab, mirikizumab	IV then SC	Risankizumab (CD, UC); mirikizumab (UC, CD)	Selectively inhibit IL-23 via the p19 subunit

Anti-TNF Agents

Anti-TNF therapy inaugurated the biologic era. An early controlled study of the chimeric monoclonal antibody cA2 (infliximab) demonstrated rapid clinical benefit in active Crohn’s disease [9], and infliximab was subsequently shown to close enterocutaneous fistulas [10]. The pivotal ACCENT I trial established that scheduled maintenance infliximab sustained response and remission and reduced corticosteroid use in luminal Crohn’s disease [11], while the ACT 1 and ACT 2 trials extended efficacy to moderate-to-severe ulcerative colitis [12]. The fully human antibody adalimumab demonstrated maintenance of remission in Crohn’s disease in the CHARM trial [13] and induction and maintenance of remission in ulcerative colitis in ULTRA 2 [14]. The landmark SONIC trial then reframed treatment strategy by showing that combination therapy with infliximab and azathioprine was superior to either agent alone in immunomodulator- and biologic-naive Crohn’s disease, providing an evidentiary basis for early, combined immunosuppression in selected patients [15]. Combination immunosuppression, however, carries safety trade-offs that should be weighed against its efficacy: the addition of a thiopurine to anti-TNF therapy increases the risk of serious and opportunistic infections and of malignancies, including non-melanoma skin cancer, lymphoma, and the rare but frequently fatal hepatosplenic T-cell lymphoma seen predominantly in young men. Treatment decisions must therefore balance the incremental efficacy of combination therapy against these risks in the individual patient.

Anti-integrin Therapy (Vedolizumab)

Vedolizumab selectively antagonizes the α4β7 integrin, interrupting the homing of gut-tropic lymphocytes to intestinal endothelium while largely sparing systemic immunity-a gut-selective mechanism with a favorable safety rationale. In the integrated GEMINI 1 (ulcerative colitis) and GEMINI 2 (Crohn’s disease) programs, vedolizumab was effective for both induction and maintenance of remission [16,17]. Its gut selectivity has made it a frequent choice for patients in whom limiting systemic immunosuppression is desirable.

IL-12/23 and Selective IL-23 Inhibition

Ustekinumab, directed against the shared p40 subunit of IL-12 and IL-23, demonstrated efficacy in Crohn’s disease in the UNITI program [18] and in ulcerative colitis in the UNIFI trial [19]. Recognition that IL-23 in particular drives pathogenic Th17 responses motivated the development of agents selective for the IL-23-specific p19 subunit. Risankizumab achieved induction of remission in the phase 3 ADVANCE and MOTIVATE trials and maintenance of remission in the FORTIFY withdrawal trial in Crohn’s disease [20,21]. Mirikizumab, another p19-selective antibody, was effective for induction and maintenance of remission in ulcerative colitis in the LUCENT program [22]. The p19-selective class continues to expand; guselkumab, for example, has more recently gained regulatory approval in ulcerative colitis and Crohn’s disease, reflecting an active pipeline of selective IL-23 inhibitors and the emergence of subcutaneous maintenance formulations that improve convenience. These selective agents have broadened the options for patients with inadequate responses to earlier therapies and have favorable tolerability profiles [23-26].

Emerging oral advanced therapies beyond biologics

The contemporary therapeutic context extends beyond injectable biologics to oral small molecules that target intracellular signaling. JAK inhibitors interrupt cytokine signaling downstream of multiple pro-inflammatory receptors: tofacitinib, a pan-JAK inhibitor, was effective for induction and maintenance of remission in ulcerative colitis in the OCTAVE program [27], and more selective agents (for example, upadacitinib) have since been approved in both ulcerative colitis and Crohn’s disease. S1P receptor modulators reduce circulating lymphocytes by sequestering them within lymph nodes; ozanimod demonstrated efficacy as induction and maintenance therapy in ulcerative colitis in the True North trial [28], and etrasimod has subsequently been approved in ulcerative colitis. These oral agents offer administration convenience and rapid onset but carry class-specific safety considerations, including, for JAK inhibitors, boxed warnings regarding thromboembolism, major adverse cardiovascular events, and malignancy in at-risk populations; and, for S1P modulators, cardiac-conduction and macular-edema monitoring requirements. Although not biologics, these therapies are integral to the changing global therapeutic landscape and to any discussion of how advanced therapy is reshaping the burden of IBD.

Comparative effectiveness and positioning

For years, the relative positioning of biologics relied on indirect comparisons and network meta-analyses because head-to-head data were scarce. The VARSITY trial-the first double-blind RCT to compare two biologics directly in IBD-demonstrated that vedolizumab was superior to adalimumab for clinical remission (31.3% versus 22.5%; a difference of 8.8 percentage points) and endoscopic improvement (39.7% versus 27.7%) at week 52 in moderate-to-severe ulcerative colitis, although it was not superior for corticosteroid-free remission [23]. In Crohn’s disease, the SEAVUE trial found ustekinumab and adalimumab to yield broadly comparable outcomes in biologic-naive patients [24]. Such trials, summarized alongside the registration studies in Table 2, increasingly inform a personalized, mechanism-aware approach to sequencing therapy. It should be emphasized, however, that validated predictive biomarkers to guide the choice of biologic for an individual patient remain limited; in current practice, treatment selection is still largely empirical, informed by disease phenotype, comorbidity, safety considerations, route preference, and cost rather than by molecular predictors of response.

**Table 2:** Selected landmark randomized controlled trials of advanced therapy in inflammatory bowel disease (IBD) Outcomes are illustrative of each trial’s principal finding; see the cited primary publications and Appendix B for full endpoint definitions and effect sizes. CD: Crohn’s disease; UC: ulcerative colitis; anti-TNF: anti-tumor necrosis factor; anti-IL: anti-interleukin; JAK: Janus kinase; S1P: sphingosine-1-phosphate.

Trial	Agent (class)	Disease	Principal finding	Ref.
ACCENT I	Infliximab (anti-TNF)	CD	Scheduled maintenance sustained response/remission and spared corticosteroids	[11]
ACT 1/ACT 2	Infliximab (anti-TNF)	UC	Induced and maintained response and remission in moderate-to-severe UC	[12]
CHARM	Adalimumab (anti-TNF)	CD	Maintained clinical response and remission	[13]
ULTRA 2	Adalimumab (anti-TNF)	UC	Induced and maintained remission in moderate-to-severe UC	[14]
SONIC	Infliximab ± azathioprine	CD	Combination superior to monotherapy in immunomodulator/biologic-naive CD	[15]
GEMINI 1/2	Vedolizumab (anti-α4β7)	UC/CD	Gut-selective induction and maintenance of remission	[16,17]
UNITI	Ustekinumab (anti-IL-12/23)	CD	Induction and maintenance of remission	[18]
UNIFI	Ustekinumab (anti-IL-12/23)	UC	Induction and maintenance of remission	[19]
ADVANCE/ MOTIVATE/FORTIFY	Risankizumab (anti-IL-23 p19)	CD	Induction (ADVANCE/MOTIVATE) and maintenance (FORTIFY) of remission	[20,21]
LUCENT	Mirikizumab (anti-IL-23 p19)	UC	Induction and maintenance of remission	[22]
VARSITY	Vedolizumab vs adalimumab	UC	Vedolizumab superior for clinical remission and endoscopic improvement at wk 52	[23]
SEAVUE	Ustekinumab vs adalimumab	CD	Comparable outcomes in biologic-naive CD	[24]
OCTAVE	Tofacitinib (JAK inhibitor)	UC	Induction and maintenance of remission (oral small molecule)	[27]
True North	Ozanimod (S1P modulator)	UC	Induction and maintenance of remission (oral small molecule)	[28]

Impact of biologics on the burden of disease

**Figure 4:**
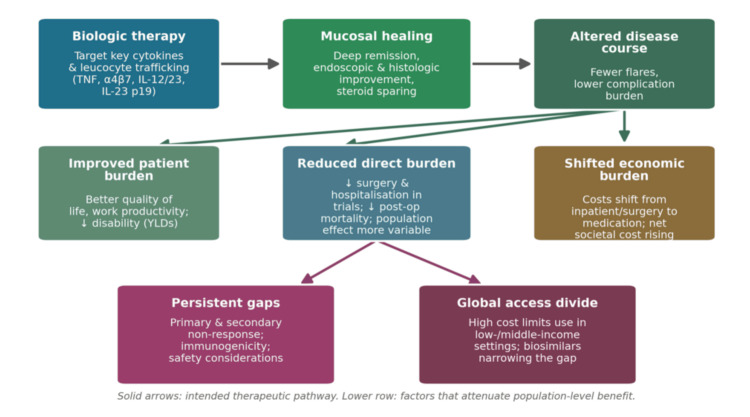
Conceptual framework for how biologic therapy may reshape the global burden of IBD. The intended pathway (upper rows) runs from mechanistic target engagement through mucosal healing to reduced complications, disability, and improved quality of life. Real-world impact is attenuated by primary and secondary non-response, immunogenicity, a shift of costs toward medication, and a persistent global access divide (lower row). This figure was created by the authors using Python version 3.10 with the Matplotlib library (Python Software Foundation, Wilmington, DE, USA).

The relevant question for global burden is not whether biologics induce remission; they demonstrably do, but whether their use translates into durable reductions in the complications, disability, and resource use that constitute the true cost of IBD. The conceptual pathway is summarized in Figure 4: by promoting mucosal healing and altering the natural history of disease, biologics are expected to reduce surgery, hospitalization, and disability while improving quality of life. The evidence, however, is more nuanced than the mechanism alone would predict. To clarify the different strands of evidence, the following subsections separate clinical and endoscopic outcomes, healthcare utilization, mortality and disability, economic burden, and global access, and throughout we distinguish evidence derived from RCTs from that derived from observational and ecological analyses.

Clinical outcomes: surgery, hospitalization, and mucosal healing

Mucosal (endoscopic) healing has emerged as a central treatment target because it correlates with reduced relapse, hospitalization, and surgery, and biologics achieve it more reliably than conventional therapy in randomized trials [8,15]. Achieving deep remission is the rationale for treat-to-target strategies that now anchor modern guidelines. Nonetheless, the population-level effect of widespread biologic use on surgical rates has been less consistent than RCT data imply, and the distinction between trial evidence and real-world observational evidence is important here. Some population-based observational cohorts report declining cumulative surgery rates in the biologic era; for example, several analyses describe reductions in early and five- to 10-year resection rates in Crohn’s disease relative to historical pre-biologic cohorts, whereas comprehensive cost-of-illness reviews caution that inpatient and surgical utilization at the population level has not fallen as anticipated [25,26]. These are observational comparisons, and the discrepancy likely reflects confounders including changing diagnostic thresholds, an enlarging and aging prevalent population, delayed access to therapy, and the inclusion of refractory patients in real-world cohorts. Quantitative estimates of the magnitude of any decline vary widely between cohorts and health systems and should be interpreted accordingly.

Mortality, disability, and quality of life

Encouragingly, age-standardized mortality from IBD has declined globally even as incidence has risen, and disability metrics suggest that effective therapy can mitigate years lived with disability [2,3]. These are, however, ecological observations derived from GBD modeling; they establish a temporal association but cannot by themselves demonstrate that biologic therapy caused the decline in mortality, which may also reflect earlier diagnosis, improved surgical and perioperative care, better management of complications, and changing disease definitions. Biologic-induced remission is consistently associated, chiefly in RCTs and their extensions, and supported by observational real-world cohorts, with improvements in patient-reported quality of life, work productivity, and corticosteroid avoidance, outcomes of major importance to a predominantly young, economically active patient population [13,19]. Because quality-of-life gains are documented in both randomized and observational settings, they are among the more robust benefits attributable to biologic therapy, whereas population-level mortality and disability trends should be read as consistent with, but not proof of, a biologic effect. Table 3 summarizes the principal domains of burden and the direction and certainty of the documented biologic effect.

**Table 3:** Domains of disease burden and the documented or expected effect of biologic therapy, with an interpretive assessment of the consistency and type of evidence. RCT: randomized controlled trial; YLD: years lived with disability

Burden domain	Effect of biologic therapy	Consistency/type of evidence
Clinical & endoscopic remission	Substantially improved versus conventional therapy across classes	High (RCT)
Corticosteroid exposure	Reduced steroid dependence and steroid-sparing effect	High (RCT)
Surgery/resection	Reduced in trials; population-level effect variable	Moderate/mixed (RCT vs observational)
Hospitalization	Reduced in trials; real-world data inconsistent	Moderate/mixed (RCT vs observational)
Mortality	Global age-standardized mortality declining	Moderate (ecological; causation not established)
Disability (YLDs) & quality of life	Improved quality of life and work productivity	High for patient-reported outcomes (RCT + observational)
Direct economic cost	Costs shift toward medication; net direct cost rising	High (cost-of-illness data)

Healthcare utilization and the economic-burden paradox

A central tension in this field is economic. Biologics are expensive, and their adoption has shifted the cost structure of IBD. Medication now constitutes the dominant component of direct expenditure. In several high-income analyses, drug costs have become the single largest category of direct cost, in some estimates approaching or exceeding half of direct expenditure, and authoritative cost-of-illness analyses indicate that total societal costs have risen rather than fallen, contrary to early expectations that reduced hospitalization and surgery would offset drug costs [26]. A balanced appraisal must, however, weigh these rising acquisition costs against potential offsetting benefits. Effective disease control can reduce hospitalizations, surgeries, and the costs of managing complications; it can also reduce the substantial indirect costs of IBD, such as absenteeism, presenteeism, reduced work productivity, and disability, which fall on patients, employers, and society and are frequently omitted from narrow drug-budget analyses. The net economic effect, therefore, depends on the perspective adopted (payer versus societal), the time horizon, and the price of the agents used, which biosimilar competition is now reducing. Thus, even where biologics increase short-term pharmaceutical expenditure, their long-term and societal economic impact is more nuanced, and the balance may shift with biosimilar-driven price reductions and with earlier, more effective disease control.

Biosimilars and the affordability of biologic therapy

Biosimilars offer the most concrete mechanism by which the population-level benefit of biologics might be extended without proportionate cost growth. The randomized, double-blind NOR-SWITCH trial demonstrated that switching from originator infliximab to the biosimilar CT-P13 was non-inferior with respect to disease worsening over 52 weeks, supporting the safety and efficacy of biosimilar substitution [25]. The entry of infliximab and adalimumab biosimilars has reduced acquisition costs substantially in many markets: in several European health systems, competitive tendering for infliximab biosimilars has lowered acquisition prices by a large margin relative to the originator (reductions on the order of 50%-80% have been reported in some national tenders), and comparable trends have followed adalimumab biosimilar entry. Where reimbursement frameworks permit and the savings are reinvested, such price reductions have expanded access and, in some systems, allowed earlier initiation of advanced therapy. Biosimilars are therefore central to any strategy that seeks to reconcile the clinical value of biologics with the financial sustainability of IBD care [26].

Global disparities and the access divide

The benefits of biologic therapy are distributed unequally. Age-standardized prevalence and the availability of advanced therapies correlate with sociodemographic development, and the steepest increases in incidence are now occurring in regions least able to finance high-cost care [3,4,5]. In many low- and middle-income settings across parts of South Asia, sub-Saharan Africa, Latin America, and the Middle East, biologics remain unavailable or unaffordable, specialist and endoscopic capacity is limited, diagnostic delay is common, and patients may present with advanced, complication-laden disease. Concrete manifestations include out-of-pocket costs for a course of originator biologic therapy that can exceed a household’s annual income, restricted formulary coverage, limited cold-chain and infusion infrastructure, and reliance on corticosteroids and thiopurines as default long-term therapy. Without deliberate policy action, including biosimilar adoption, pooled or centralized procurement, capacity building for diagnosis and infusion services, and the integration of IBD into universal health coverage, the global divergence in outcomes is likely to widen even as the science advances [26]. The paradox of the modern era is that the tools to reduce the burden of IBD exist but are inaccessible to a large share of the patients who need them most.

Future directions

Several developments may further reshape the burden of IBD. First, the proliferation of mechanistic classes, together with emerging oral small molecules such as JAK inhibitors and S1P receptor modulators, supports a move toward a precision-medicine approach in which therapy is matched to the dominant inflammatory pathway in the individual patient; it should be acknowledged, however, that validated predictive biomarkers are not yet available for routine use, and such individualization remains largely aspirational rather than established clinical practice. Second, head-to-head and treat-to-target trial designs, exemplified by VARSITY and SEAVUE, will continue to refine optimal positioning and sequencing [23,24]. Third, the maturation of the biosimilar market and the development of subcutaneous formulations may improve both affordability and convenience [25]. Finally, realizing population-level benefit will require investment in early diagnosis, predictive biomarkers of response, implementation science, and equitable access so that the demonstrable efficacy of biologics is translated into measurable reductions in surgery, disability, and premature mortality across all regions [5,26].

Limitations

This narrative review has limitations. It is selective rather than systematic, and it therefore reflects the authors’ interpretation of a large and rapidly evolving literature; relevant studies may have been omitted, and no formal risk-of-bias assessment or quantitative synthesis was performed. Because we prioritized landmark trials and high-impact publications, the review is also subject to publication bias: pivotal industry-sponsored RCTs with positive results are more likely to be published, published prominently, and cited than negative, inconclusive, or unpublished studies, which may bias the apparent balance of evidence toward benefit. Epidemiological estimates derived from modeled GBD data carry uncertainty, particularly in regions with sparse primary data, and different GBD cycles revise historical estimates, so figures from different cycles are not directly comparable. Crucially, much of the population-level evidence linking biologics to reduced mortality and disability is ecological or observational; such data cannot establish direct causation, and the observed trends likely reflect multiple contributing factors beyond biologic therapy. Efficacy data are drawn predominantly from regulatory RCTs conducted in selected populations, which may not generalize to real-world practice. Finally, the field is advancing quickly; newer agents, indications, and long-term comparative data continue to emerge and may modify the conclusions drawn here.

## Conclusions

Biologic therapies have transformed the treatment of moderate-to-severe IBD, delivering reliable induction and maintenance of remission, mucosal healing, corticosteroid sparing, and meaningful improvements in quality of life across an expanding range of mechanistic classes. Against a backdrop of a globalizing and growing prevalent population, these agents have plausibly contributed to declining age-standardized mortality and reduced disability for treated patients; this contribution, however, is inferred largely from ecological and observational data and cannot be regarded as proof of direct population-level causation, since improved diagnosis, surgical care, and complication management also play a part. Their effect on the aggregate global burden of IBD is therefore incomplete and uneven. Population-level reductions in surgery and hospitalization have been less consistent than trial data would predict, the cost of care has shifted toward medication and risen overall, and access remains starkly inequitable between high-income and resource-limited settings. Biologics are therefore best understood not as a solution to the global burden of IBD in themselves but as powerful tools whose population impact will be determined by biosimilar-enabled affordability, optimal positioning within treat-to-target strategies, early diagnosis, and equitable access. Closing the gap between what biologics can achieve for the individual and what they achieve for populations is the central challenge of the next decade, and it is as much a challenge of affordability and implementation science as of drug discovery.

## Appendices

Appendix A 

**Table 4:** Literature search strategy

Element	Detail
Databases	MEDLINE/PubMed; supplemented by reference lists of identified articles, major society guidelines, and Global Burden of Disease publications
Date span	Inception to the final search date in May 2026; no lower date limit applied to landmark trials
Language	English-language publications
Core search terms	(“inflammatory bowel disease” OR “Crohn disease” OR “ulcerative colitis”) AND (biologic* OR “tumor necrosis factor” OR infliximab OR adalimumab OR golimumab OR certolizumab OR vedolizumab OR ustekinumab OR risankizumab OR mirikizumab OR “interleukin-23” OR integrin OR biosimilar) AND (epidemiology OR “global burden” OR incidence OR prevalence OR surgery OR hospitali* OR “quality of life” OR cost OR remission OR “randomized controlled trial”)
Study types prioritized	Phase 2/3 RCTs underpinning regulatory approval; population-based epidemiological analyses; Global Burden of Disease analyses; head-to-head comparative trials; authoritative systematic reviews and consensus statements
Screening process	Records screened independently by two authors against the prioritization hierarchy; disagreements resolved by discussion and adjudication by a senior author. A formal PRISMA flow diagram and record counts were not maintained, consistent with the narrative design
Exclusion	Non-English reports; conference abstracts without peer-reviewed publication; studies superseded by later, more complete reports
Selection approach	Interpretive selection by the authors with emphasis on landmark and high-impact evidence; no formal risk-of-bias scoring or quantitative synthesis was performed

Appendix B 

**Table 5:** Design summary of pivotal trials. Note: VARSITY reported week-52 clinical remission in 31.3% of vedolizumab-treated versus 22.5% of adalimumab-treated patients, and endoscopic improvement in 39.7% versus 27.7%, respectively; corticosteroid-free remission did not differ significantly between groups [23]. Readers are directed to the primary publications for complete numerical results for all other trials. CD: Crohn’s disease; UC: ulcerative colitis; anti-TNF: anti-tumor necrosis factor; anti-IL: anti-interleukin; JAK: Janus kinase; S1P: sphingosine-1-phosphate.

Trial	Disease	Enrolled (n)	Design / primary endpoint focus	Ref.
ACCENT I	CD	573	RCT; maintenance of clinical response/remission after infliximab induction	[11]
ACT 1 / ACT 2	UC	See ref.	Two RCTs; clinical response and remission with infliximab	[12]
CHARM	CD	See ref.	RCT; maintenance of response and remission with adalimumab	[13]
ULTRA 2	UC	494	RCT; induction and maintenance of remission with adalimumab	[14]
SONIC	CD	508	RCT; corticosteroid-free remission with infliximab ± azathioprine vs monotherapy	[15]
GEMINI 1	UC	See ref.	Integrated induction/maintenance RCT; vedolizumab	[16]
GEMINI 2	CD	See ref.	Integrated induction/maintenance RCT; vedolizumab	[17]
UNITI / IM-UNITI	CD	See ref.	Induction and maintenance RCT program; ustekinumab	[18]
UNIFI	UC	See ref.	Induction and maintenance RCT; ustekinumab	[19]
ADVANCE / MOTIVATE	CD	See ref.	Phase 3 induction RCTs; risankizumab (IL-23 p19)	[20]
FORTIFY	CD	See ref.	Phase 3 randomized withdrawal maintenance RCT; risankizumab	[21]
LUCENT 1 / LUCENT 2	UC	See ref.	Phase 3 induction and maintenance RCTs; mirikizumab (IL-23 p19)	[22]
VARSITY	UC	769	Head-to-head double-blind RCT; vedolizumab vs adalimumab; wk-52 clinical remission	[23]
SEAVUE	CD	See ref.	Head-to-head RCT in biologic-naive CD; ustekinumab vs adalimumab	[24]
NOR-SWITCH	Multiple	See ref.	Randomized non-inferiority switch trial; originator infliximab vs CT-P13	[25]
OCTAVE (Induction 1/2, Sustain)	UC	See ref.	Phase 3 induction and maintenance RCTs; tofacitinib (JAK inhibitor)	[27]
True North	UC	See ref.	Phase 3 induction and maintenance RCT; ozanimod (S1P modulator)	[28]
